# Gaucher’s Disease

**Published:** 1916-06

**Authors:** 


**Gaucher's Disease.** J. H. M. Knox, H. Roswell Wahl and H. C. Schmeisser, Johns Hopkins Hosp. Bull. Jan., give a table of the 16 cases already known. Age limits 14 months to 44 years; sex 3 male: 13 female; splenomegaly up to 45x25x13 c.m., weight 8000 grams, averaging 1/5-1/6 of body weight instead of the normal 1:400; blood as in secondary anaemia, leukopenia down to 500 per c.m.m. In 12 cases, histories suggesting the same disease in brothers or sisters were noted. Splenectomy was done in 9 cases, 3 dying shortly after, the others living at least for a few weeks, up to 16 months and apparently nothing further being reported as to survival. Syphilis and tuberculosis seem to have nothing to do with the development of the disease though one instance of the former and 3 of the latter are reported in the 16 cases. The characteristic lesion, on which the definition depends, is the presence of peculiar pale round or oval cells with an average diameter of 20-40 microns, especially in the spleen, also in the liver, lymph nodes, bone marrow and in the authors' cases quite generally distributed. The authors add two cases, sisters, aged respectively 9 and 5 months, the 11th and 12th children, 6 other children, 20-4 years old (plus 2 years to date of second case) well, four dead, one at 8th month of unknown cause the rest of various causes, not suggesting Gaucher's disease. Both cases died. The pathologic findings are reported in detail.

